# Expression of an expanded CGG-repeat RNA in a single pair of primary sensory neurons impairs olfactory adaptation in *Caenorhabditis elegans*

**DOI:** 10.1093/hmg/ddu210

**Published:** 2014-05-12

**Authors:** Bi-Tzen Juang, Anna L. Ludwig, Kelli L. Benedetti, Chen Gu, Kimberly Collins, Christopher Morales, Aarati Asundi, Torsten Wittmann, Noelle L'Etoile, Paul J. Hagerman

**Affiliations:** 1 Department of Biological Science and Technology, National Chiao Tung University, Hsinchu 300, Taiwan,; 2 Department of Biochemistry and Molecular Medicine, University of California, Davis, School of Medicine, Davis, CA 95616, USA,; 3 Department of Cell and Tissue Biology, University of California, San Francisco, San Francisco, CA 94143, USA; 4 MIND Institute, University of California, Davis, Health System, Sacramento, CA 95817, USA

## Abstract

Fragile X-associated tremor/ataxia syndrome (FXTAS) is a severe neurodegenerative disorder that affects carriers of premutation CGG-repeat expansion alleles of the fragile X mental retardation 1 (*FMR1*) gene; current evidence supports a causal role of the expanded CGG repeat within the *FMR1* mRNA in the pathogenesis of FXTAS. Though the mRNA has been observed to induce cellular toxicity in FXTAS, the mechanisms are unclear. One common neurophysiological characteristic of FXTAS patients is their inability to properly attenuate their response to an auditory stimulus upon receipt of a small pre-stimulus. Therefore, to gain genetic and cell biological insight into FXTAS, we examined the effect of expanded CGG repeats on the plasticity of the olfactory response of the genetically tractable nematode, *Caenorhabditis elegans* (*C. elegans*). While *C. elegans* is innately attracted to odors, this response can be downregulated if the odor is paired with starvation. We found that expressing expanded CGG repeats in olfactory neurons interfered with this plasticity without affecting either the innate odor-seeking response or the olfactory neuronal morphology. Interrogation of three RNA regulatory pathways indicated that the expanded CGG repeats act via the *C. elegans* microRNA (miRNA)-specific Argonaute ALG-2 to diminish olfactory plasticity. This observation suggests that the miRNA-Argonaute pathway may play a pathogenic role in subverting neuronal function in FXTAS.

## INTRODUCTION

The molecular pathogenesis of the fragile X family of disorders involves expansions of a non-coding CGG-repeat microsatellite in the 5′ untranslated region (5′UTR) of the fragile X mental retardation 1 (*FMR1*) gene (1–4). Fragile X syndrome, the most common heritable form of intellectual disability and most common single-gene form of autism, is associated with CGG-repeat expansions that exceed 200 repeats (full mutation), almost always accompanied by epigenetic silencing (5). Repeat expansions in the 55–200 range (premutation) give rise to the neurodegenerative disorder, fragile X-associated tremor/ataxia syndrome (FXTAS) (6–8), and to the reproductive disorder, fragile X-associated primary ovarian insufficiency (9,10). The repeat is also associated with early onset attention and intellectual deficit disorders (2,11).

FXTAS is a progressive movement disorder seen in older males in or beyond the sixth decade in life (2,12,13), with the preponderance of evidence to date indicating a pathogenic mechanism involving ‘toxicity’ (functional cellular impairment) of the expanded CGG-repeat element within the *FMR1* mRNA (2,14). Several observations have led to the hypothesis that the premutation-allele message is likely to be the key pathogenic agent causing cellular toxicity in FXTAS. First, intranuclear inclusions containing the expanded CGG-repeat *FMR1* mRNA in post-mortem brain tissue have been observed in FXTAS patients (15). Similar intranuclear inclusions and neuronal cell death were observed in Purkinje neurons in a mouse model that expressed premutation CGG repeats upstream of an enhanced green fluorescent protein (GFP) reporter in the absence of the *FMR1* gene (16). Second, intranuclear inclusions were observed in neural cell culture following overexpression of a premutation-length CGG element upstream of a GFP reporter (17,18). However, inclusions were not found in the same model in the absence of transcription, implicating the message and not CGG-repeat DNA in inclusion formation. Third, recent evidence suggests that (i) sequestration of one or more proteins by the CGG repeat (as RNA) leads to a functional deficiency of those proteins (19,20), and that (ii) expression of additional proteins may be dysregulated by microRNAs (miRNAs) that target the *FMR1* mRNA (21). These results have established a linkage between the expansion of the premutation CGG-repeat of *FMR1* mRNA and FXTAS (17). Finally, premutation alleles are associated with a substantial increase (2- to 8-fold) of *FMR1* mRNA (22,23); thus, RNA toxicity may arise through increased CGG-repeat length or increased mRNA expression, or both (24). *FMR1* transcript levels were also increased in mouse models of FXTAS, up to 6-fold in brain, when the mouse endogenous CGG repeat was replaced by premutation-length CGG repeats (25–27).

While CGG-repeat-expressing mammalian cell and animal models are able to reproduce the formation of intranuclear inclusions, as well as the increased levels of expanded CGG-repeat mRNA, cell culture systems cannot capture the intricacy of complex neural circuits in an intact organism, nor can they probe non-cell-autonomous effects only produced within an organism, such as aging and metabolism, on neural function. Moreover, an additional CGG-repeat toxicity model, *Drosophila melanogaster*, is limited because the affected neurons in the eye-expression model die in substantial numbers, an outcome not seen in other FXTAS models. Further, working with mouse models requires significant time and expense to create and study the appropriate phenotypes, thus preventing the use of these animal models in high-throughput screens for genetic pathways that may either enhance or suppress the phenotype.

Here we have established a nematode (*Caenorhabditis elegans, C. elegans*) premutation model, which has a well-defined and genetically tractable neuronal circuit, and exhibits robust behavioral plasticity. The model offers an attractive alternative to the mouse or fly models with which to explore the cellular and molecular mechanisms underlying the neuronal pathologies induced by expression of expanded (99 CGG) *FMR1* repeats. Additionally, though *C. elegans* is an invertebrate, we share ∼83% of the same proteins (28), and its simple nervous system, comprised of 302 neurons, is capable of producing complex, malleable behaviors. In particular, the odor-seeking behavior of the nematode is simple in that it is generated by just four olfactory sensory neurons [two pairs of AWA and amphid wing cells AWC neurons for attractive olfactory behaviors] (29), and yet it is complex in that the attractiveness of an odor is modified by experience (30,31). Indeed, a single AWC neuron senses butanone and causes naïve nematodes to move toward this odor source (32), and prolonged odor exposure in the absence of food reduces the animal's attraction to butanone (30,31). We have shown that this behavioral plasticity results from processes that occur within the primary odor-sensory (AWC) neuron (33–37).

To model FXTAS in *C. elegans*, we developed transgenic animals that expressed a GFP reporter containing the human *FMR1* 5′UTR either without any CGG repeats (0CGG), with an intermediate number of repeats (16CGG or 30CGG), or with 99 CGG repeats (99CGG), driven by an AWC-specific promoter. Here we find that the plasticity of the response to butanone is impaired in animals that express 99 CGG repeats. However, olfactory plasticity is not affected by expression of the control *FMR1* 5′UTR lacking CGG repeats or expressing the intermediate number of repeats. The reduced neuronal plasticity seen in the 99 CGG-repeat lines is reminiscent of the reduced pre-pulse inhibition seen in FXTAS patients (38). Finally, we show that the microRNA-specific Argonaute ALG-2 is required for the decreased plasticity of animals expressing expanded repeats in the AWC neurons.

## RESULTS

### Expression of a premutation CGG-repeat expansion in the 5′UTR of a GFP reporter results in loss of olfactory adaptation in *C. elegans*

The *C. elegans* AWC sensory neurons allow worms to track and pursue (chemotax toward) attractive volatile chemical stimulants and provide an exquisitely sensitive and specific neuronal model for assessing the effects of CGG-repeat-induced RNA toxicity; the neurons govern both the primary olfactory response and a secondary adaptive response, which requires neuronal plasticity (36,39). Moreover, *C. elegans* provides a powerful system for genetic screens aimed at exploring the mechanism of CGG-repeat-induced RNA toxicity. To assess the sensitivity of AWC neurons to expanded CGG-repeat RNA, we first determined whether expression of a premutation CGG-repeat expansion affected either the primary (chemotaxis) or secondary (adaptive or reduced chemotaxis) olfactory responses.

**Figure 1.**
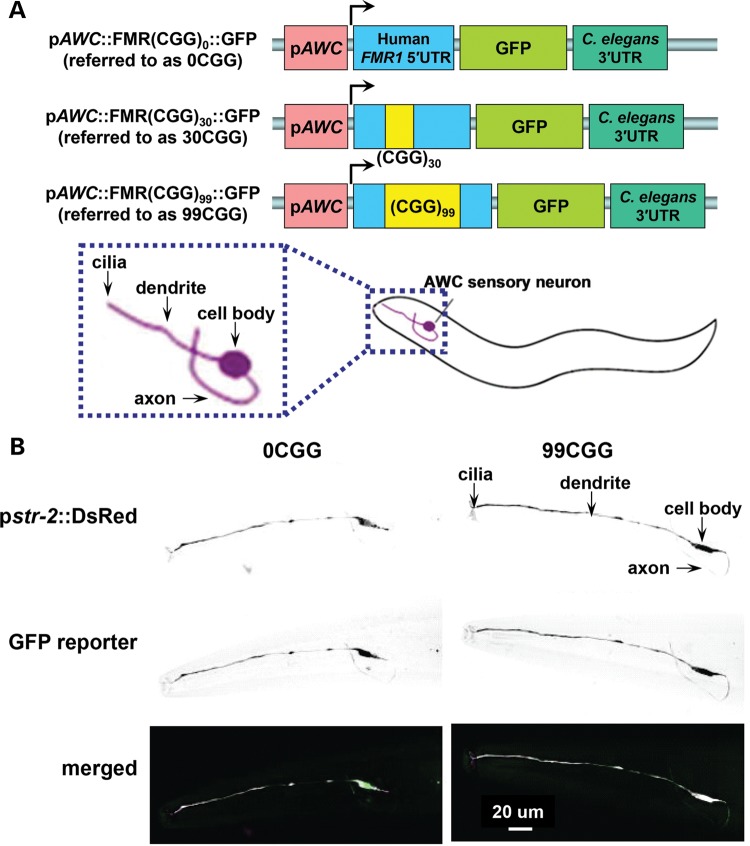
Expression of an expanded CGG-repeat reporter in AWC neurons. (**A**) Representation of the GFP reporter containing expanded CGG repeats. GFP reporters, carrying a human *FMR1* 5′UTR fragment with 0, 30, or 99 CGG repeats, were expressed under an AWC-specific promoter (p*AWC*); these reporters are referred to as 0CGG, 30CGG and 99CGG*.* The open reading frame for GFP was followed by a 3′UTR element from *unc-54*. Arrow denotes the start site of transcription. (**B**) Expression of the GFP reporter in the AWC neuron. (Top) Schematic diagram of the AWC neuron in the head of *C. elegans*. Inset represents the area visualized in confocal florescence images below. The anatomy of the AWC neuron is diagramed in an enlarged cartoon in the left dotted box. (Bottom) Confocal florescence images of transgenic animals expressing GFP in the AWC neuron. GFP is expressed either from the *FMR1* 5′UTR without CGG repeats (left) or with 99 CGG repeats (right). The AWC neuron was identified by expression of DsRed from an AWC-specific promoter (p*str-2*) and the anatomy is labeled in the 99CGG animal. Anterior is left for both images.

Human genomic sequences encoding the *FMR1* 5′UTR, with either 0 or 99 CGG repeats, were sub-cloned into a low-copy plasmid (pBR322) upstream of a GFP reporter sequence (Fire lab vector, pPD95.75). These sequences were placed transcriptionally downstream of the AWC-specific promoter p*ceh36*^prom3^ (referred to herein as p*AWC*) (40). A 3′ untranslated region (3′UTR) from *unc-54*, a standard *C. elegans* 3′UTR used in transgene expression in somatic cells (41), was inserted downstream of the GFP coding sequence. The resulting reporter is designated p*AWC*::FMR(CGG)_99_::GFP (referred to as 99CGG in all figures; Fig. 1A). As a control for transgene expression, the CGG-repeat element was removed from the 5′UTR; the control plasmid was designated p*AWC*::FMR(CGG)_0_::GFP (referred to as 0CGG in all figures; Fig. 1A). These GFP reporters were injected into wild-type animals with the AWC^ON^ marker, p*str-2*::DsRed, and the co-injection marker, p*unc-122*::GFP, which is expressed in coelomocytes. GFP expression in the AWC neuron could be visualized in these transparent transgenic animals (Fig. 1B), and transgenic worms carrying either the 0CGG or 99CGG element were observed to express GFP throughout each AWC neuron. Importantly, the morphology of the neurons was not altered when the p*AWC*::FMR(CGG)_99_::GFP was expressed (compare 0CGG to 99CGG in Fig. 1B).

**Figure 2.**
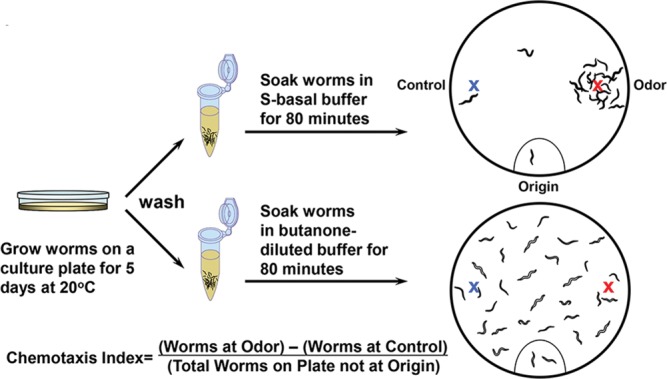
Scheme of olfactory adaptation. Adult animals were washed free from bacteria, then half of the population was exposed to buffer alone (naïve animals; top) and the other half exposed to buffer with diluted butanone (pre-exposed animals; bottom). After 80 min incubation, animals were placed at the ‘origin’ of a 10 cm assay plate with a butanone spot (red ‘X’) and a control ethanol spot (blue ‘X’). Animals roamed for 2 h at 20°C, after which their olfactory behavior was quantified by the chemotaxis index (CI).

An initial screen for chemotaxis was conducted to determine whether expression of the 0CGG or 99CGG transgene affects olfactory behavior. The olfactory assay was performed as shown in Figure 2. Approximately 200 adult worms from an individual transgenic line were split into two populations; one group was exposed to S-basal buffer alone (to test for chemotaxis), while the other was pre-exposed to the AWC-sensed odor, butanone, in S-basal buffer for 80 min under otherwise identical conditions (to test for adaptation). After removing the odorant by washing the worms with S-basal buffer, animals were placed on an assay plate, with an ethanol-diluted butanone point source opposite from an ethanol point source, and were allowed to roam for 2 h at 20°C. Olfactory behavior was quantified in terms of the chemotaxis index (CI): the number of animals in a defined area near the attractive odor (point source) minus the number of animals in an equivalent area near the ethanol point source; the difference is divided by the total worms on the assay plate, excluding the origin area (39). A CI of 1 indicates strong attraction to the odor (primary response; chemotaxis), while a CI of 0 indicates the absence of any attraction to the odorant (secondary response; adaptation). For wild-type animals, prolonged odor pre-exposure led to olfactory adaptation and reduced CI to less than one half of the value of the naïve animals exposed to S-basal buffer alone. Using these assays, transgenic animals could be analyzed for both primary and adaptive responses to odorant.

**Figure 3.**
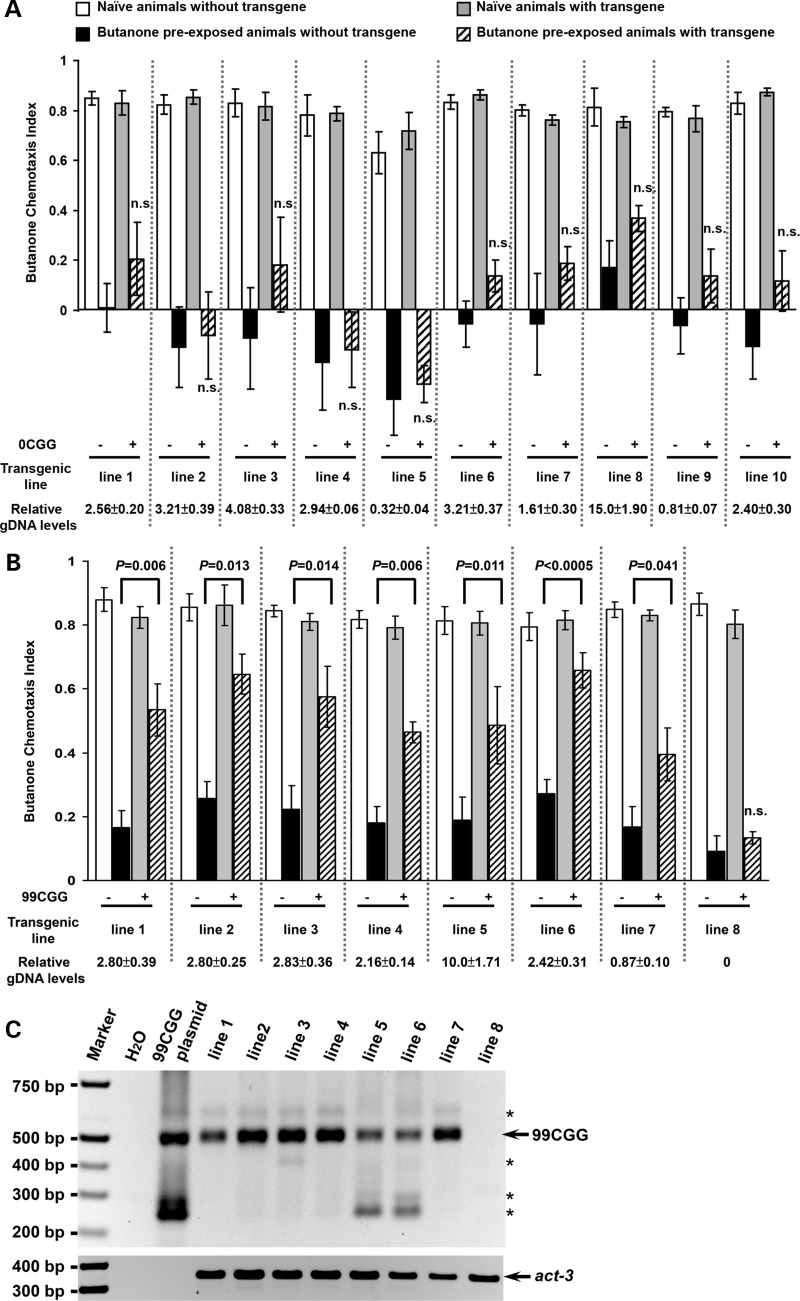
Expression of 99 CGG repeats in the 5′UTR results in olfactory adaptation defects. (**A**) Animals carrying the control GFP reporter with 0 CGGs in the 5′UTR behave like wild type. Transgenic strains (‘+’; express the *FMR1* reporter and p*unc-122*::GFP in coelomocytes) and siblings that lost the transgene (‘−’) were grown on the same plates. Bars and error bars represent the mean CIs and standard errors of the mean (SEM) from at least 3 independent assay days from populations that were either naïve (white or gray bars) or pre-exposed to odor (black or hatched bars). To quantitate the relative gDNA levels, total gDNA from the larval stage 4 (L4) animals was extracted and subjected to real-time PCR. The relative levels of the 0CGG and 99CGG reporters were normalized to the housekeeping gene, *act-3*. The data were collected from three independent experiments. ‘±’ indicates the values of SEM. (**B**) 100% of lines that express 99 CGG repeats are defective for olfactory adaptation. The chemotaxis and adaptation values for seven transgenic lines (lines 1–7) carrying the p*AWC*::FMR(CGG)_99_::GFP construct (labeled (+)) were compared with their siblings without transgenes (labeled (−)). Data are from at least five independent assays and are labeled as in Figure 3A. *P*-values indicate results of two-tailed *t*-tests between the CIs of transgenic- (+) and non-transgenic (−) adapted animals in the same transgenic strain. ‘n.s.’ indicates no significant difference, as *P*-value is >0.05. The relative gDNA levels are presented at the bottom line. (**C**) PCR genotyping of transgenic animals carrying 99 CGG repeats. gDNA from individual strains was extracted and genotyped with two primers flanking the CGG-repeat area. The PCR product is 505 bp, indicated by an arrow, and the p*AWC*::FMR(CGG)_99_::GFP plasmid, referred as CGG plasmid, was used as a positive control. Amplification of an endogenous control, *act-3*, is shown below. ‘*’ indicates non-specific amplification. The absence of a band for line 8 indicates that this line had lost the *FMR1* reporter.

Ten transgenic strains expressing p*AWC*::FMR(CGG)_0_::GFP from independent injections all behaved like their sibling wild-type animals for both chemotaxis (exposure to S-basal buffer alone; Fig. 3A, gray bars) and adaptation to butanone (butanone-diluted S-basal buffer exposure; hatched bars). Siblings without transgenes also had normal behaviors for both chemotaxis (Fig. 3A, white bars) and adaptation responses (black bars). The wild-type behavior of p*AWC*::FMR(CGG)_0_::GFP-expressing lines demonstrated that the *FMR1* 5′UTR element without CGG repeats, though highly-GC-rich (∼74% GC) relative to the *C. elegans* genome (∼36% GC) (42), did not appreciably affect either primary or adaptive responses to an AWC-sensed odor.

Eight transgenic strains expressing p*AWC*::FMR(CGG)_99_::GFP were produced by separate injections. The transgenic animals and their non-transgenic siblings displayed similar attraction to butanone (Fig. 3B, white and gray bars). However, when we analyzed adaptation, we found that seven out of eight transgenic strains were impaired in their ability to adapt to butanone and remained attracted to the odor point source (Fig. 3B, hatched bars); a Student's two-tailed *t*-test was performed to examine the difference between the adapted responses of the transgenic and non-transgenic siblings. This finding was in contrast to their sibling strains, which had lost the transgene and exhibited normal plasticity in response to prolonged butanone pre-exposure (Fig. 3B, black bars). To determine whether the absence of a behavioral difference in the one adaptive line (line 8) was due to loss of p*AWC*::FMR(CGG)_99_::GFP sequences, we performed polymerase chain reaction (PCR) analysis. Genomic DNA (gDNA) from individual strains was isolated, and the crude extracts were genotyped by PCR amplification through the CGG-repeat element (43). Figure 3C shows that the p*AWC*::FMR(CGG)_99_::GFP transgene was lost in line 8. Thus, expression of the expanded repeat reduced neuronal plasticity in *C. elegans*. These lines were also tested for their ability to chemotax toward and adapt to the two other AWC-sensed odors, benzaldehyde and isoamyl alcohol. We found that naïve chemotaxis was unaffected, but adaptation to benzaldehyde, as with butanone, was impaired in animals that expressed 99CGG (Supplementary Material, Fig. S1).

The penetrance of the adaptation defects was 100% since all lines that expressed p*AWC*::FMR(CGG)_99_::GFP were impaired for adaptation. To determine whether behavioral plasticity was affected by the GC ‘load’ on the animals, we examined the copy number of each transgene by PCR of the *FMR1* 5′UTR-GFP junction. When *C. elegans* gonads are injected with plasmid DNA, they package the concatenated DNA into an extrachromosomal array that is maintained like an extra X chromosome. Each line will have a unique number of transgenes within the array. This number is stable over the generations, though the array itself can be lost. When gDNA levels from each line were normalized to the endogenous housekeeping gene *act-3*, we found that the relative gDNA levels mostly ranged from 2 to 4 (Fig. 3A and B), and yet the adaptation defects of the 99CGG line 5, which carries 10-fold more transgenes, was no more adaptation-defective than line 6 (Fig. 3B). We chose to integrate the extrachromosomal DNA of line 6 p*AWC*::FMR(CGG)_0_ and line 6 p*AWC*::FMR(CGG)_99_ into the genome, as this facilitates strain maintenance and allowed us to have a stable baseline for the 0CGG and 99CGG analysis that would follow. Importantly, each line had comparable numbers of transgenes prior to and after integration (0CGG: 3.2/2.5 transgenes/10^2^ *act-3,* before/after integration; 99CGG: 2.4/1.9 transgenes/10^2^ *act-3,* before/after integration).

### CGG-repeat length has a greater effect on plasticity than copy number

**Figure 4.**
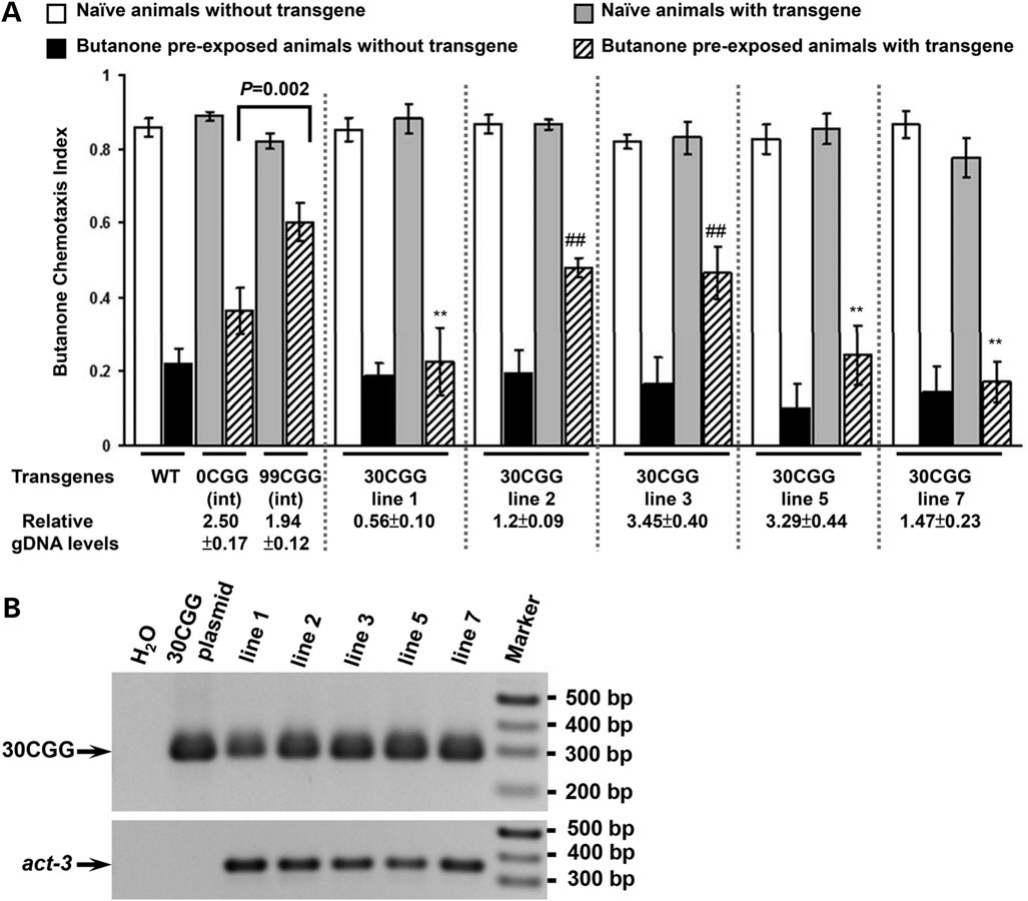
Animals that express 30 CGG repeats exhibit mild and variable adaptation defects. (**A**) Bars indicate the CI of lines that express 30 CGG repeats in the *FMR1* 5′UTR (last five sets of bars), when compared with wild type, and the integrated lines expressing 0 or 99 CGG repeats in the *FMR1* 5′UTR (first three sets of bars). The relative gDNA levels shown below each line were calculated by normalizing reporter levels to the housekeeping gene, *act-3.* gDNA was collected from three separated experiments. Student *t*-tests between the adapted values for animals that expressed the reporter (hatched bars) and animals that express 0CGG repeats showed no significant differences. Comparison between the adapted CIs of the transgenic animals (hatched bars) and those of their non-transgenic siblings (black bars) indicate that 2/5 lines (lines 2 and 3) showed significant differences, which are marked with #. Comparison between the adapted CIs of the transgenic animals (hatched bars) and the CIs from the 99CGG-expressing line (sixth bar) showed that the adaptation behavior of lines 1, 5 and 7 were significantly different from the adaptation behavior of the 99CGG-expressing line. The *P*-values <0.005. ‘##’ indicates two-tailed *t*-test showing *P* < 0.005 between the adapted CI of animals with transgene and without transgene. Data were collected from at least 5 independent assay days. Error bars present SEM. (**B**) PCR genotyping of transgenic animals. 298 bp of PCR fragments were amplified in animals carrying 30CGG repeats in the 5′UTR. The lower panel shows the amplified fragments from the endogenous gene, *act-3*.

To understand which affected plasticity more, the copy number or repeat length, we constructed 5 lines expressing extrachromosomal arrays with 30 CGG repeats and examined their behavior (Fig. 4A). We also tried to obtain lines with 16 CGG repeats but we could only generate two lines, too small a number to use for assessment of penetrance. The behavior of the 16 CGG-repeat-expressing lines is shown in Supplementary Material, Figure S2A. When we examined the 30 CGG-repeat-expressing lines, we found that none (0/5) showed significantly higher adaptation values than those seen in the integrated line with 0CGG repeats. In contrast to the 0 CCG-expressing lines, 2/5 30 CGG-repeat-expressing lines (lines 30-2 and 30-3) had adapted CIs that were significantly higher than their non-transgenic siblings. The adapted CIs of these strains lay midway between the CI values of the 0 and 99 CGG integrated strains and were not statistically different from either. Thus, the penetrance and severity of the adaptation defects correlates with the number of repeats such that 99 CGGs produce a very penetrant (100%) and moderate to severe adaptation defect, and the normal mode for human repeats (30) produces a mild defect that is only 40% penetrant in *C. elegans*. When we examined the copy number and asked whether the repeat length or the copy number of the transgenes has a greater effect on adaptation, we found that repeat length affects the penetrance of the adaptation defect more than copy number. That is, 100% of the 99 CGG-expressing lines were impaired for adaptation, though their relative copy numbers varied from 1 (line 7) to 10 (line 5) transgenes/10^2^ act-3 genes (Fig. 3B). The penetrance of the adaptation defects was 40% in the lines with 30 CGGs, and the value of the adapted CIs did not correlate with the copy number of the transgenes (Fig. 4A). That is, two lines with 30 CGGs (lines 3 and 5) had nearly identical copy numbers (3.45 versus 3.29 genes/10^2^ *act-3*, respectively), but line 3 had mild adaptation defects, while line 5 behaved like wild type. Importantly, all lines retained the same number of repeats (see genotyping gel in Fig. 4B). Thus, repeat size is more important than copy number, which rules out an ectopic CGG RNA-specific response.

### *FMR1* mRNA levels for 99CGG lines are increased over 0CGG in *C. elegans*

**Figure 5.**
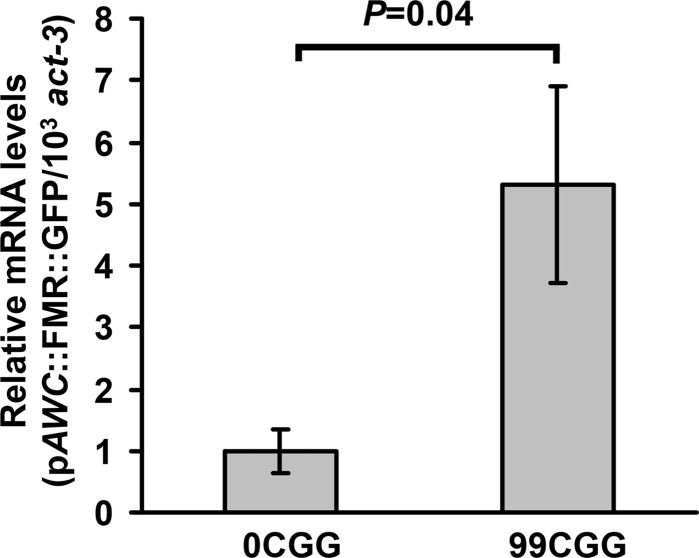
The 5′UTR CGG repeat causes a significant increase in mRNA level. Total RNA from adult transgenic animals was extracted, reverse-transcribed into cDNA, and quantified by real-time PCR. GFP mRNA expression was increased ∼5-fold in animals expressing p*AWC*::FMR(CGG)_99_::GFP compared with control (0CGG) animals, which was set to 1. (Error bars: SEM.) The data were collected from four independently isolated populations of animals.

We previously showed that *FMR1* mRNA levels in premutation individuals are elevated 2- to 8-fold over normal (22,23). Likewise, *FMR1* mRNA levels in cell and animal models expressing the premutation CGG-repeat element in the 5′UTR increased 2- to 6-fold compared with a lower number of repeats (17,25–27,44). To determine whether the level of mRNA in *C. elegans* is similarly increased in animals that contain 99 CGG repeats, we performed quantitative reverse transcriptase PCR analyses of total mRNA from each line. First, we integrated the transgene into the genome of each strain. We chose to integrate lines of 99CGG and 0CGG with similar transgene copy numbers. The primers used in our analyses amplified a region from the very 3′ end of the *FMR1* 5′UTR to the 5′ portion of the GFP-coding region. Expression was normalized to the housekeeping gene, *act-3*. We found that the level of mRNA containing 99 CGG repeats was elevated by ∼5-fold over that of the control reporter gene (Fig. 5). Thus, by this second metric (mRNA level), expression of the 99 CGG-repeat allele in the *C. elegans* olfactory neuron mirrors what is observed in human patients with FXTAS.

### Expression of the 99CGG transgene does not affect the morphology or the cell fate of the AWC neuron

**Figure 6.**
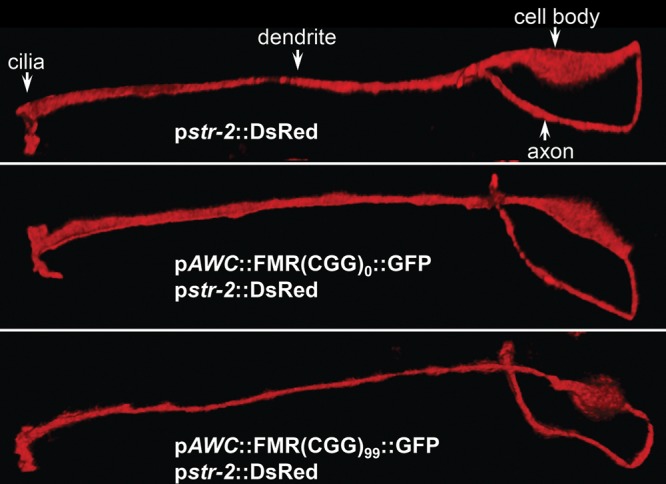
The expanded CGG repeats in the 5′UTR of the GFP reporter do not affect the morphology or the cell fate of the AWC neuron. The morphology of the AWC neuron was examined by observing expression of p*str-2*::DsRed. The shape of the neuron was identical in wild-type (top) and transgenic animals with 0CGG (middle) or 99CGG (bottom). Anatomy of the AWC neuron is indicated in the top panel.

Murine hippocampal neurons cultured from mice with premutation CGG repeats showed elevated *FMR1* mRNA and neurotoxicity phenotypes, such as decreased viability and dendritic complexity, as well as changed synaptic morphology (45). To determine whether expression of expanded CGG repeats might affect the structural integrity of the AWC neurons, we expressed the AWC^ON^ p*str-2*::DsRed reporter in double-transgenic lines, which showed diffuse red florescence throughout the neuron. No obvious change in morphology was observed in naïve animals expressing either p*AWC*::FMR(CGG)_0_::GFP (Fig. 6, middle) or p*AWC*::FMR(CGG)_99_::GFP (bottom) compared with the wild-type animal alone (top). Note that the morphology of the 99 CGG-expressing AWC may be rounder in this image than the 0 CGG-expressing neuron, but this cell shape is polymorphic and does not represent a true deviation from the normal shape. Indeed, in Figure 1B, the cell shapes are very similar.

Although both AWC olfactory neurons have similar structures and functions, chemosensory receptor STR-2 is randomly expressed in either the left or right AWC neuron in wild-type animals, termed the AWC^ON^ neuron (46). AWC^ON^ senses the odor butanone and can promote either attractive or repulsive behaviors (32). In some cases, mutations that cause STR-2 to be expressed in both AWC neurons also cause the animal to fail to adapt to butanone (32,47). To understand whether expressing 99CGG alters AWC cell fate, we examined STR-2-driven DsRed fluorescence in wild-type, 0CGG and 99CGG on 3 separate days. Asymmetric expression of STR-2 was observed in all strains and on each day, with 93% of transgenic animals with the 99 CGG-repeat element in the 5′UTR of the GFP reporter displaying asymmetric STR-2 expression (Table 1). Therefore we conclude from these initial studies that the reduced olfactory plasticity seen in animals that express 99CGG is not due to changes either in cellular morphology or cell fate.

**Table 1.** Asymmetric expression of str-2 in AWC neurons^^a^^

Genotype	Percentage of animals with			*n*
	0 AWCp*str-2* ON	1 AWCp*str-2* ON	2 AWCp*str-2* ON	
Wild type	3	97	0	112
Wild type + FMR(CGG)_0_	0	100	0	129
Wild type + FMR(CGG)_99_	5	93	2	120

^a^Cell fate of the AWC neuron was examined by quantitating the asymmetric expression of p*str-2*::DsRed. Data were collected from three independent lines, and animals were scored by three categories according to pst*r-2*::DsRed expression in neither AWC (0 AWC p*str-2* ON), in only one AWC (1 AWC p*str-2* ON), or in both AWC (2 AWC p*str-2* ON).

### Identifying genes that interact genetically with 99CGG to interfere with neuronal plasticity

Although RNA toxicity imparted by premutation CGG-repeat elements is believed to be the pathogenic basis of FXTAS, downstream pathways by which this RNA effect is mediated remain obscure. To begin to address this issue, we examined three RNA-processing pathways required in AWC neurons to promote adaptation, and asked whether their adaptation defects could be modified by expression of 99 CGG repeats. Strains integrated with p*AWC*::FMR(CGG)::GFP were used for this investigation of candidate genetic modifiers.

**Figure 7.**
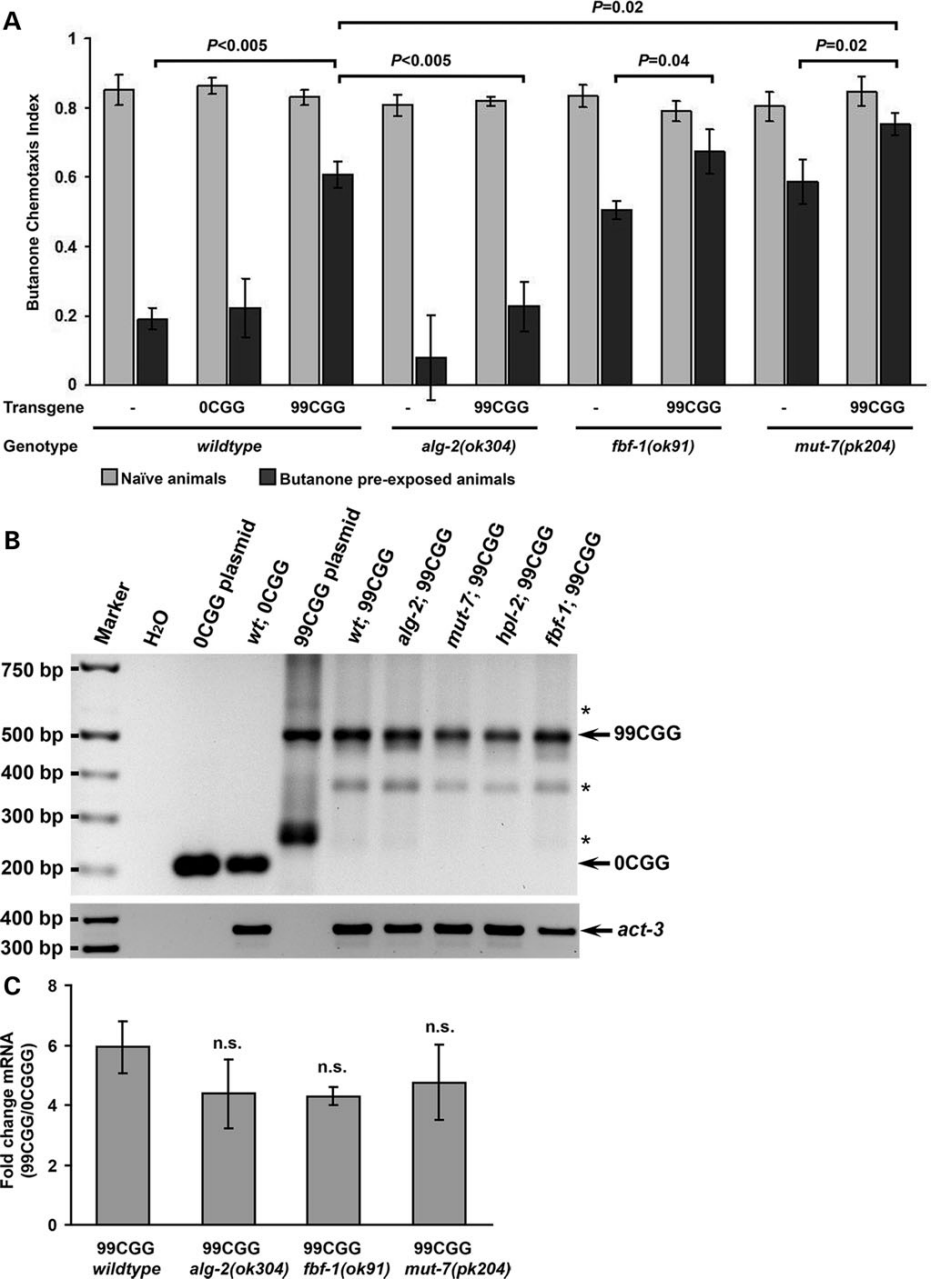
Interactions between the premutation CGG repeats and adaptation-promoting pathways. (**A**) Olfactory adaptation. 99CGG animals were crossed with mutants defining three pathways: ALG-2 is an miRNA-specific Argonaute, FBF-1 up-regulates EGL-4 translation, and MUT-7 is required for siRNA processing. *alg-2* knockout decreased the effect of expanded CGG repeats, reducing adaptation defects in the double mutants. Conversely, the adaptation defects of 99CGG animals crossed with *fbf-1* and *mut-7* mutants were partially additive to defects in 99CGG animals alone. CI experiments were performed in at least triplicate, and error bars represent SEM. (**B**) Genotyping of animals with transgenes. 206- and 505-bp PCR fragments were amplified in animals carrying p*AWC*::FMR(CGG)_0_::GFP and p*AWC*::FMR(CGG)_99_::GFP plasmids. A few non-specific bands are indicated by ‘*’. (**C**) Expanded-repeat mRNA levels had no significant change after crossing with *alg-2*, *fbf-1*, and *mut-7* knockout lines. Bars represent the fold change of FMR(CGG)_99_ mRNA levels in p*AWC*::FMR(CGG)_99_::GFP, or in either *alg-2*, *fbf-1* or *mut-7* double mutants, respectively, compared to the mRNA levels of a control p*AWC*::FMR(CGG)_0_::GFP. The data were collected from four independent experiments.

The cGMP-dependent protein kinase EGL-4 is necessary for olfactory adaptation of the AWC neuronal response (36), and butanone adaptation requires increased translation of *egl-4* mRNA, which is facilitated by the RNA-binding protein, FBF-1 (34). Strains that lack *fbf-1* are adaptation defective because they fail to up-regulate the kinase EGL-4. FBF-1 is a member of the Pumilio/Fem-3 binding factor (PUF) family that binds to the *egl-4* 3′UTR and enhances its translation (34). We asked whether expression of the p*AWC*::FMR(CGG)_99_::GFP alters the adaptation defects resulting from loss of FBF-1. To this end, the integrated p*AWC*::FMR(CGG)_99_::GFP strain was crossed with an *fbf-1* null mutant. We found that the adaptation defects of the *fbf-1(ok91*) strain were slightly, though significantly, increased when they expressed p*AWC*::FMR(CGG)_99_::GFP [CI (adapted) of *fbf-1* = 0.51, 99CGG = 0.61, *fbf-1* and 99CGG = 0.67; two-tailed Student's *t*-test between *fbf-1* and *fbf-1/*99CGG; double mutant *P* = 0.04] (Fig. 7A), although the *fbf-1(ok91)* mutant that expressed 99 CGG repeats in the 5′UTR did not exhibit altered STR-2 cell fate (Table 2). These olfactory behavioral results raise the possibility that the premutation expansion of the *FMR1* gene may partially reduce the translational enhancement of EGL-4 through interference with FBF-1 function.

**Table 2.** str-2 expression in AWC neurons (integrated)

Genotype	Percentage of animals with			*n*
	0 AWCp*str-2* ON	1 AWCp*str-2* ON	2 AWC*pstr-2* ON	
Wild type	1	99	0	90
Wild type + FMR(CGG)_0_	0	100	0	90
Wild type + FMR(CGG)_99_	0	100	0	90
*alg-2(ok304)* + FMR(CGG)_99_	2	98	0	90
*fbf-1(ok91)* + FMR(CGG)_99_	0	100	0	90
*mut-7(pk204)* + FMR(CGG)_99_	0	100	0	90

### The endogenous RNAi pathway is partially affected by expression of the premutation repeats

Recently, we reported that the endogenous nuclear interference RNA (RNAi) pathway (48) is required in AWC for olfactory adaptation (33). The endogenously-produced small RNAs that we found to be required for odor adaptation are amplified from mRNA by an RNA-dependent RNA polymerase; therefore, these 22-nt RNAs are antisense to the original template. The RNAs are bound by the Argonaute NRDE-3, which shuttles them into the nucleus where they base pair with the nascent strand of mRNA, stall Polymerase II and guide repressive chromatin complexes to the gene by which they are encoded. In this way, endogenous siRNAs allow a gene to silence itself. We found that the nuclear RNAi and the FBF-1-mediated adaptation pathways function in parallel, such that double mutants are completely adaptation defective. To understand whether 99CGG expression interferes with RNAi-mediated adaptation, we turned to the RNAi defective strain, *mut-7(pk204)*. MUT-7 is a putative 3′-5′ exonuclease required for transposon (49) and transgene (50) silencing, as well as for nuclear RNAi maturation (51); this strain is adaptation defective (33). When we introduced the expanded CGG-repeat strain into a *mut-7* null mutant strain (49), we found that expression of the fragile X premutation in *mut-7(pk204)* animals led to significantly worse adaptation defects than seen either in wild-type animals expressing a CGG-repeat element, or in the *mut-7(null)* alone [CI (adapted) of *mut-7(pk204)* and 99CGG = 0.75; *mut-7(pk204)* = 0.59, *P* = 0.02; 99CGG = 0.61, *P* = 0.02] (Fig. 7A). However, the *mut-7(pk204)* mutant with the integrated 99 CGG repeat exhibited normal STR-2 cell fate (Table 2). These behavioral results suggest that the expanded repeats partially interfere with a MUT-7-driven adaptation process. Nevertheless, pathways in addition to RNAi are likely to be affected by the expanded repeat. HPL-2 is a counterpart of the human heterochromatin binding protein 1 (HP1) and acts in the same genetic pathway with MUT-7 (33). However, when we expressed the expanded repeat in an *hpl-2* null mutant, *hpl-2(tm1489)*, the animals were sterile (data not shown). Importantly, in each genetic background mentioned above, the integrated repeats were the same length and copy number (Fig. 7B).

### Toxicity of the expanded repeat requires an intact miRNA pathway

Several studies have indicated that miRNAs are involved in neuronal development and in learning and memory formation (52). In *C. elegans*, canonical miRNA biogenesis requires DRSH-1, the Drosha RNase III-type ribonuclease, and its binding partner, PASH-1 (53). PASH-1 is a counterpart of DGCR8 in human and Pasha in fly, and acts with DRSH-1 to process primary (pri)-miRNAs in the nucleus. The pri-miRNA is then exported to the cytoplasm where RNAse III DCR-1 recruits miRNA-specific Argonaute (AGO) proteins, such as ALG-1 and ALG-2 (54), to further process pri-miRNA into mature miRNA. These miRNAs repress translation of target mRNAs, and they may also have nuclear functions (55). In addition to DROSHA/PASHA-dependent miRNAs, several other small non-coding RNAs, including small nucleolar RNA (snoRNA) and spliced intronic RNAs, can be exported from the nucleus and processed into miRNAs; these latter RNA species are termed mitrons and siRNAs (56).

To determine whether the neurotoxicity of the expanded repeats acts via an miRNA-dependent process, we introduced the integrated p*AWC*::FMR(CGG)_99_::GFP into an *alg-2* (*ok304*) null mutant strain (57). ALG-2 is one of two *C. elegans* miRNA Argonautes, and the primary species expressed in neurons (58). We observed that loss of this Argonaute restored adaptation to strains that expressed the fragile X premutation CGG repeat (CI = 0.23), compared with the adaptation defects seen in wild-type animals that express the same transgene (CI = 0.61, *P* < 0.005) (Fig. 7A). Once again, STR-2 cell fate is unaltered in these strains (Table 2).

In *C. elegans*, ALG-2 associates with another miRNA specific Argonaute, ALG-1, in a DCR-1 complex in the cytoplasm to produce mature miRNA (59). Unfortunately, due to the small brood size of the *alg-1* null mutant strain, we were unable to perform behavioral assays.

Genotyping analysis showed that the transgenic array was invariant between all the strains we assessed (Fig. 7B). The rescued behavior was not due to a reduction of repeat-containing RNA in *alg-2* null animals, as the levels of mRNA produced from the expanded repeat were the same in each strain (Fig. 7C). Thus, ALG-2 is likely to be required to cause the adaptation defects resulting from expression of the fragile X premutation CGG repeat in AWC.

## DISCUSSION

FXTAS is a human, progressive neurodegenerative disorder in which the abnormal CGG expansion within the 5′UTR of the *FMR1* gene is thought to be the source of abnormal cell function. Although expression of premutation CGG-repeat alleles in cell and animal models has recapitulated several features of the cellular and behavioral phenotypes of FXTAS, including intranuclear inclusions and mRNA accumulation, the molecular basis of the relationship between the CGG-triplet expansion and progressive cognitive and behavioral difficulties remains unclear. Here we report a *C. elegans* model for premutation-driven dysfunction, and demonstrate that 99 CGG repeats (near the modal value for the repeat length among FXTAS patients) in the 5′UTR of a GFP reporter lead to defects in neuronal plasticity when expressed solely in an olfactory neuron. The defect is independent of neuronal development, as cell fate and morphology were not affected.

**Figure 8.**
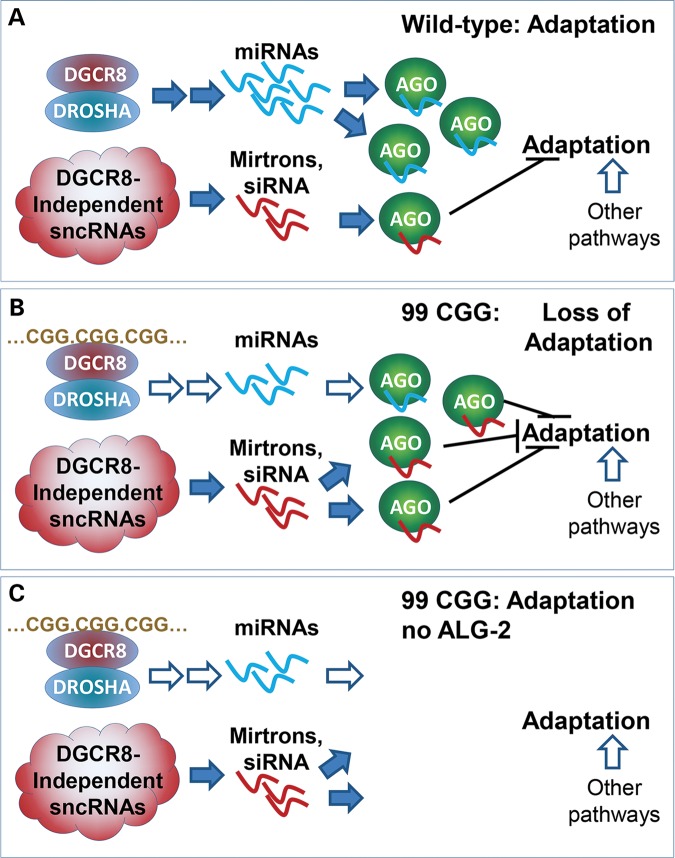
A speculative model for the rescue of adaptation by loss of the Argonaute, ALG-2. (**A**) In the wild-type animal, DROSHA/DGCR8 produces pre-miRNAs (labeled as blue squiggles), that do not interfere with adaptation. We postulate this because *drsh-1* and *alg-2* mutants are wild type for adaptation (33) (Supplementary Material, Fig. S1). One or more mirtrons/miRNAs produced from DROSHA/DGCR8-independent pathways (red squiggles) may negatively regulate adaptation, but normally play a minor role. (**B**) Once the 99CGG RNA sequesters DGCR8, blue miRNA expression is repressed and the balance between blue and red (adaptation neutral and adaptation repressive) microRNAs is tipped, with AGO-bound repressive mirtron/miRNAs blocking adaptation. (**C**) In the absence of ALG-2, the inhibition of the adaptation response is relieved, since neither class of microRNA is active without an Argonaute.

This impairment of behavioral plasticity in adult animals carrying 99 CGG repeats provides a model with which to examine how the 5′UTR premutation CGG expansion causes changes in behavior. In addition, our analysis of pathways that require RNA processing for adaptation shows that the CGG-repeat element is likely to interfere with plasticity via an miRNA pathway, since loss of miRNA-specific Argonaute ALG-2 restores behavioral plasticity. MicroRNAs, small non-coding RNAs that are not translated into proteins, have been implicated in the regulation of many cellular and organismal processes, including learning and memory (60). One speculative model for how loss of the Argonaute ALG-2 might suppress adaptation is presented in Figure 8. In the absence of an expanded CGG repeat (wild type), DROSHA/DGCR8 makes the bulk of the pre-miRNAs, which are processed into the bulk of miRNAs in the cell. These miRNAs are not required for adaptation, since loss of DROSHA (DRSH-1) or the microRNA Argonaute, ALG-2, does not affect adaptation (33) (Supplementary Material, Fig. S1). We suggest that among the small fraction of miRNAs that are DROSHA/DGCR8-independent, there may be individual miRNAs that negatively regulate adaptation, but which would normally not have much of an effect. However, once DGCR8 is bound by 99CGGs and canonical miRNA production is repressed (20), the relative fraction of mirtrons and other DGCR8-independent, small non-coding RNAs (sncRNAs) would increase. Babiarz *et al*. (56) show that the levels of many DGCR8-independent miRNAs increase in dgcr8−/− mouse ES cells, and Sellier *et al*. (20) showed that the mirtron levels are not diminished. We speculate that the ALG-2-bound mirtrons and other DGCR8-independent miRNAs that repress genes required for adaptation are more active as they have no competition for ALG-2 and thus much of the ALG-2 function is redirected to minor targets; targets might include genes that are required for adaptation, such as OSM-9 and EGL-4. Consequently, loss of ALG-2 would relieve inhibition that arises from the DGCR8-independent miRNAs repressing their targets. This would indicate that a set of DGCR8-independent miRNAs can alter neuronal function in the context of 99CGG expression.

Biochemical studies of mouse and fly models of FXTAS show that many RNA-binding proteins, including those required for miRNA biogenesis and function, interact with premutation CGG repeats. Two lines of evidence indicate that miRNAs, or their associated factors, may play important roles in the pathogenic action of the expanded repeat. First, in the FXTAS fly model, miRNA-277 (miR-277) was found to affect CGG-mediated neuronal toxicity by regulating expression of the DNA fragmentation factor-related protein 2 (Drep-2) and the visceral mesodermal armadillo-repeats (Vimar) at the mRNA level (61). Second, in the brains of individuals who died with FXTAS, the miRNA-processing, double-stranded RNA-binding protein, DGCR8, and its partner, DROSHA, were shown to associate with premutation CGG repeats (20). This association sequestered pri-miRNAs and decreased levels of many miRNAs. Sellier *et al*. (20) proposed that the CGG-mediated decrease in free DGCR8 and DROSHA, and the attendant reduction in key miRNAs, caused loss of neuronal dendritic complexity and cell viability. It remains to be determined whether the DGCR8 homolog, Pasha, interacts with the CGG repeat, and is therefore functionally sequestered, in the same manner as in humans, or whether other proteins are functionally impaired by the CGG-repeat element.

Our candidate approach revealed that miRNA-binding Argonaute ALG-2 is likely to be required for CGG-repeat-induced defects in olfactory adaptation. ALG-2 appears to be the primary neuronal Argonaute in *C. elegans* head neurons (58). In other tissues, ALG-2 can be associated with miRNA Argonaute ALG-1. These Argonaute proteins and miRNA are found in a complex with DCR-1, a Dicer ribonuclease III. It will be interesting to investigate whether loss of other ALG-2-associated factors likewise protects neurons from the toxic effects of expanded CGG repeats. One particularly compelling interaction is between ALG-2 and AIN-1, since this interaction implicates a *C. elegans* Sam68 homolog; Sam68 sequestration has been suggested as a participant in the pathogenesis of FXTAS (62).

During development of *C. elegans* somatic tissues, the Argonaute binding partner, GW182 AIN-1, interacts with the core miRNA RNA-induced silencing complex (miRISC) components ALG-1 and ALG-2 (63). This core machinery also associates with a TRIM-NHL protein, NHL-2, and a DEAD-Box protein, CGH-1, to enhance translational repression (64). In addition to translational repression, CGH-1 and GLD-1 (an RNA-binding protein) also mediate mRNA stabilization in nematode oocytes (65). GLD-1 has a KH1 RNA-binding domain, which shows high similarity with Sam68. In the cytoplasm, GLD-1 is able to associate with AIN-2, a homolog of AIN-1 and an miRISC (66). Thus, loss of ALG-2 from this complex may reduce the sequestration of Sam68 or PASH-1 (the *C. elegans* DGCR8 homolog), thereby releasing these factors and allowing them to perform their important cellular functions.

Though sequestration is likely to play a dominant role in the neuropathology of CGG repeats in *C. elegans* neurons, as in both human and other animal models, it is possible that other modes of toxicity could be operating as well, as in the repeat-associated non-ATG (RAN) translation mechanism proposed by Todd *et al*. (67). However, it remains to be determined whether RAN products contribute to the neurological phenotype in FXTAS. In this regard, it is noteworthy that the ‘NIH’ FXTAS mouse line of Usdin and co-workers (26,68,69), which does not produce RAN products, exhibits a neurodegenerative phenotype, whereas the ‘Dutch’ FXTAS mouse line, which is capable of generating RAN products, does not have a significant neurodegenerative phenotype (2).

The processes of releasing neurotransmitters are sensitive to downstream cellular changes. Studies of FXTAS mice demonstrated increased mRNA levels in GABAergic neurons (70). Cell culture from hippocampal neurons of male FXTAS mice displayed imbalanced excitotoxicity from activation of glutamate receptors and inhibition of GABA receptor (71). The AWC sensory neuron is glutamatergic (72), and this nematode system may be able to clarify the regulation of glutamate-mediated transmission and aspects of plasticity caused by the premutation CGG repeats.

Finally, the finding that loss of ALG-2 ameliorates all the effects of the premutation repeat means that therapies based on the ability to inactivate an Argonaute or an Argonaute-mediated event might be designed. It would be particularly interesting to identify a drug target such as a kinase that is involved in regulation of the function of this Argonaute.

## MATERIALS AND METHODS

### Worm strains

The *C. elegans* Bristol N2 was used as a wild-type strain in this study; mutant alleles include *alg-2*(*ok304*), *fbf-1*(*ok91*), *hpl-2*(*tm1489*), and *mut-7*(*pk204*). Strains were maintained at 20°C using standard protocols (73). All transgenic lines were created by injecting 1 ng/µl of the constructs into wild-type N2 animals, along with co-injection markers, which included coelomocyte marker p*unc-122::GFP* (20 ng/µl) and AWC marker p*str-2::*DsRed (20 ng/µl). Transgenes were integrated into the nematode genome by trimethylpsoralen (TMP) (74). Transgenic animals at L4 stage were exposed to TMP and ultraviolet, and ∼100 fraction of first generation progeny animals were clonally expanded. After hatching, ∼500 filial generation were randomly clonally expanded. The integrants were screened by 100% transmission of co-injection marker p*unc-122::*GFP. The integrated strains were outcrossed with wild-type animals at least three times to eliminate TMP-induced mutations in the genome. The integrated strain, carrying 99 CGG repeats in the 5′UTR of the GFP reporter, was crossed with mutant strains, including *alg-2*(*ok304*), *fbf-1*(*ok91*), *hpl-2(tm1489)* and *mut-7*(*pk204*). The individual genotypes were confirmed by either sequencing for *mut-7*(*pk204*) with a point mutation near the 3′ end, or by PCR for DNA deletions of *alg-2*(*ok304*), *fbf-1*(*ok91*) and *hpl-2(tm1489)*.

### Plasmid construction

An AWC-specific promoter from a truncated form of the *ceh-36* promoter, termed p*ceh-36^prom3^*, was inserted into pPD95.75 using PstI and BamHI upstream of GFP, referred to as p*AWC*::GFP (a kind gift from Oliver Hobert (40)). Before inserting the 5′UTR of the *FMR1* sequence, the plasmid was first changed to a low-copy plasmid with the origin of replication (Ori) in reverse orientation (opposite direction as the transcript) to ensure that CGG repeats do not delete during *Escherichia coli* propagation (75). pBR322 (76) was digested with *Pvu*II and *Hin*dIII, and the larger fragment (2324 bp) containing the Ori and ampicillin-resistance gene was purified. p*AWC*::GFP was digested with *Hin*dIII and *Ssp*I, and the smaller fragment (1475 bp) containing the promoter and the GFP-coding region was ligated to the Ori fragment (*Ssp*I and *Pvu*II digest to blunt ends), making a p*AWC*::GFP-low-copy plasmid. Next, to facilitate insertion of *FMR1* 5′UTRs, a linker (5′- gatccccgggtcaggcgctcagcatccgttattggaagctagcagggctgaagagaacggtac), containing a *Blp*I site at the 5′ end and an *Nhe*I site at the 3′ end of the *FMR1* 5′UTR, was inserted between the *AWC* promoter and GFP with *Bam*HI and *Kpn*I digestion. The 5′UTR fragment of *FMR1* containing either 0 or 99 CGG repeats was obtained from *FMR1* 5′UTR(0 CGG)-FL and *FMR1* 5′UTR(99 CGG)-FL (77) by cutting with BlpI and NheI, and was inserted into the engineered p*AWC*::GFP, which was digested with the same restriction enzymes to create p*AWC*::FMR(CGG)_0_::GFP and p*AWC*::FMR(CGG)_99_::GFP plasmids. An *unc-54* 3′UTR element was required for stably expressing GFP in *C. elegans*; thus, this fragment was amplified from pPD95.75 using two primers (5′-TGGAAACATTCTTGGACACAA and 5′-tattctgtcatttaagtatacGGCAAAAACCCCATAGACAC containing an AccI recognition site, and a few extended bases (lower case) required for efficient enzyme cleavage). The PCR product was digested with MfeI and AccI, and was then inserted downstream of GFP in the p*AWC*::FMR(CGG)_0_::GFP and p*AWC*::FMR(CGG)_99_::GFP constructs, which had been cut with the same restriction enzymes.

### Isolation of gDNA from worms and CGG-repeat genotyping

Total gDNA from each transgenic strain was extracted in single worm lysis buffer (50 mm KCl, 10 mm Tris-Cl pH 8.0, 2.5 mm MgCl_2_, 0.45% NP40, 0.45% Tween-20, and 60 µg/ml proteinase K). The mixture was frozen at −80°C for 10 min, followed by incubation at 60°C for 1 h, and proteinase K was inactivated at 95°C for 15 min. Using a modification of a PCR protocol (43), animals were genotyped for the expanded CGG repeat by using the Expand Long Template PCR System (Roche Diagnostics Corporation, Indianapolis, IN, USA). PCR of either 20 ng genomic or 2 ng plasmid DNA was performed in a 30-µl reaction with 2.25 m betaine (Affymetrix, Santa Clara, CA, USA), 500 µm deoxyribonucleotide triphosphates (dNTP) (GeneAmp, Life Technologies, Grand Island, NY, USA), 0.33 µm of each primer (MWG-Biotech AG, Huntsville, AL, USA) and 1 U DNA polymerase using 10× buffer 2 (both are included in PCR kit). The primer sequences are 5′-TCAGGCGCTCAGCTCCGTTTCGGT and 5′-TCTACCGGTACCGTTCTCTTCAGCCCTGCTAGC. The amplification cycling was as follows: initial denaturation at 98°C for 10 min, 10 cycles of 97°C for 35 s, 62°C for 35 s and 68°C for 4 min; then 25 additional cycles in which the elongation step is increased by 20 s every cycle, and a final elongation at 68°C for 10 min. To prevent non-specific amplification, the reaction was allowed to denature for 8 min before the polymerase was pipetted into each reaction. Amplification of the endogenous actin gene was performed as follows: either 20 ng genomic or 2 ng plasmid DNA, and 0.42 mm dNTP (GeneAmp), 0.33 µm each primer, 1.25 U Amplitaq Gold (Life Technologies), 1.5 mm MgCl_2_ and 3.0 µl 10× PCR Gold buffer were combined in a final volume of 30 µl. The primer sequences are *act-3* forward primer 5′-cggtatgggacagaaggac and a reverse primer 5′-ggaagcgtagagggagagga. Reactions were denatured at 95°C for 1 min, cycled 30 times at 95°C for 15 s, 60°C for 15 s and 72°C for 1 min, followed by a final elongation at 72°C for 2 min. A portion of each reaction was then run next to the HiLo DNA Marker (Bionexus, Inc., Oakland, CA, USA) on a 0.9% agarose gel, and was imaged according to standard procedures.

### mRNA quantitation

Two 10 cm plates of adult animals were harvested by washing with S-Basal and split into two populations: ∼50 animals were lysed in 40 µl of single worm lysis buffer for gDNA preparation, as described above; the remaining animals were frozen at −80°C for total RNA preparation. Total RNA was extracted by Trizol reagent (Life Technologies) and miRNeasy micro kit (Qiagen, Valencia, CA, USA), and then treated with Turbo DNase I (Ambion, Life Technologies) at 37°C for 20 min for removing contaminated gDNA. One microgram of total RNA was used in a cDNA synthesis reaction with iScript cDNA Synthesis Kit (Bio-Rad Laboratories, Hercules, CA, USA). Two microliters of cDNA was used in real-time PCR with Brilliant III Ultra-Fast SYBR Green qPCR Master Mix (Agilent Technologies, Santa Clara, CA, USA) containing 200 nm of forward and reverse primers (5′-cagggctgaagagaacggta and 5′-cgagaagcattgaacaccataa) in a 20 µl reaction volume. This amplicon includes sequences downstream of the CGG-repeat element and extending to the first 267 bp of GFP gDNA. The qPCR reaction mixtures were prepared in triplicate for each sample and thermocycling conditions were carried out on a Bio-Rad Chromo4 system, with denaturation at 95°C for 10 min, followed by 40 cycles of 20 s at 95°C, and 20 s at 60°C. All reactions showed a single dissociation curve, which indicated specific amplification.

The mRNA expression level of each CGG-GFP reporter was first normalized to the mRNA of the housekeeping gene, *act-3*; *act-3* mRNA was amplified by the primers: ggttgccgctcttgttgtag and accgaccatgactccttgat. The final CGG-mRNA expression per gDNA was calculated by taking the respective ratio of the mRNA to the value of gDNA normalized to the *act-3* DNA; qPCRs were performed by using 2 µl of gDNA extract under the same conditions.

### Copy-number analysis

gDNA from 20 animals at larval stage 4 (L4) was extracted in 40 µl of single worm lysis buffer, as described above. For 0CGG and 99CGG analysis, 2 µl of gDNA was used in real-time PCR with Brilliant III Ultra-Fast SYBR Green qPCR Master Mix (Agilent Technologies), and the samples were prepared as described above. Thermocycling was carried out on a Stratagene Mx3000P instrument with denaturation at 95°C for 10 min, followed by 40 cycles of 15 s at 95°C and 1 min at 60°C. For 16CGG and 30CGG analysis, 2 µl of gDNA was mixed with SensiFAST SYBR Lo-ROX mix (Bioline), and thermocycling conditions were performed on a Viia7 qPCR machine (Life Technologies) with denaturation at 95°C for 3 min, followed by 40 cycles of 5 s at 95°C, and 30 s at 60°C. The relative gDNA levels were calculated by normalizing to the *act-3* gDNA.

### Behavioral assays

Behavioral assays were performed as described in Colbert and Bargmann (30). Briefly, L4 animals were grown on NGM agar plates (2.5 g peptone, 17 g agar, 3 g NaCl per 1 l) seeded with bacterial strain HB101 as animals' food at 20°C for 5–6 days; adults were collected by washing with S-basal buffer [5 g NaCl and 50 ml of 1 m potassium phosphate (pH 6.0) per liter] and equally transferred into two unautoclaved 1.5 ml microcentrifuge tubes. After three washes and sedimenting the worms by gravity, half of the animals were pre-exposed to 1.5 ml diluted butanone (11 µl butanone in 100 ml S-basal), and the other half were incubated with S-basal buffer as a control population. Pre-exposure was carried out for 80 min at 20°C on a Labquake rotor (Thermo Fisher Scientific, Inc., Waltham, MA, USA). Animals were washed twice with S-basal and once with water, then the animals were placed on 10 cm chemotaxis assay plates (10 ml of 1.6% agar in 5 mm potassium phosphate (pH 6.0), 1 mm CaCl_2_ and 1 mm MgSO_4_). During the washes, 1 µl of 1 m sodium azide was applied to two odorant spots on the assay plates. After transferring ∼100 worms to the assay plates, 1 µl diluted butanone (1:1000 dilution in ethanol) was applied to an odorant spot on an assay plate, while 1 µl 100% ethanol was applied opposite to the odorant spot. Animals on the assay plates were allowed to move for 2 h at 20°C and then their positions were scored. Transgenic animals were scored under the fluorescence-dissecting scope using co-injection marker p*unc-122*::GFP. Each experiment was conducted through at least three separate assays, with >100 animals per assay.

Olfactory behavior was quantified by calculating the CI, which is calculated by subtracting the number of animals at the ethanol spot from the number at the odor, and dividing this sum by the number of animals that left the origin.

## SUPPLEMENTARY MATERIAL

Supplementary Material is available at *HMG* online.

## FUNDING

This work was supported by the National Institutes of Health (R01 HD040661 to P.J.H.; R01 DC005991 to N.D.L.; and R25 GM56847 graduate fellowship to K.L.B.). Funding to pay the Open Access publication charges for this article was provided by a grant from the National Institutes of Health (R01 DC005991 to N.D.L.).

## Supplementary Material

Supplementary Data
