# Supplementary material for: Expression of an expanded CGG-repeat RNA in a single pair of primary sensory neurons impairs olfactory adaptation in Caenorhabditis elegans
Source: Hum Mol Genet. 2014 May 12;23(18):4945–59. doi: 10.1093/hmg/ddu210 (PMC4140470; doi:10.1093/hmg/ddu210)
Supplement: Supplementary Data [file supp_23_18_4945__index.html]

Expression of an expanded CGG-repeat RNA in a single pair of primary sensory neurons impairs olfactory adaptation in Caenorhabditis elegans — Expression of an expanded CGG-repeat RNA in a single pair of primary sensory neurons impairs olfactory adaptation in Caenorhabditis elegans — Supplementary Data 

# Expression of an expanded CGG-repeat RNA in a single pair of primary sensory neurons impairs olfactory adaptation in *Caenorhabditis elegans*

## Supplementary Data

Supplementary Data

**Files in this Data Supplement:**

- Supplementary Data - Pdf file
