# Supplementary material for: Expression of an expanded CGG-repeat RNA in a single pair of primary sensory neurons impairs olfactory adaptation in Caenorhabditis elegans
Source: Hum Mol Genet. 2014 May 12;23(18):4945–59. doi: 10.1093/hmg/ddu210 (PMC4140470; doi:10.1093/hmg/ddu210)
Supplement: Supplementary Data [file supp_ddu210_ddu210supp.pdf]

Figure S1. Analysis of the adapted response to different odorants. Animals carrying 0CGG or 99CGG transgenic arrays were pre-exposed to different AWC-sensed odors including butanone, benzaldehyde, and isoamyl alcohol. Two-failed t-test was performed by comparing CIs of adapted animals carrying 0CGG and 99CGG from at least three independent assays in each odor exposure.

Figure S2. The olfactory behavior of animals expressing 16 CGG repeats in the 5′UTR of the GFP reporter. (**A**) Transgenic animals carrying 16 CGG repeats in the 5′UTR behave similarly to the 0CGG integrated animals. "#" indicates that the CIs of adapted animals with transgene compared with non-transgene show significant difference ($P<0.05$). Each bar shows the mean from at least three independent experiments and error bar represents SEM. The average relative genomic DNA levels of transgenic animals from three independent experiments are quantified by real-time PCR and normalized to the housekeeping gene *act-3*. (**B**) PCR genotyping of transgenic animals. 256 bp of PCR fragments were amplified in animals carrying 16 CGG repeats in the 5′UTR. Each endogenous *act-3* amplification is showed in the panel.

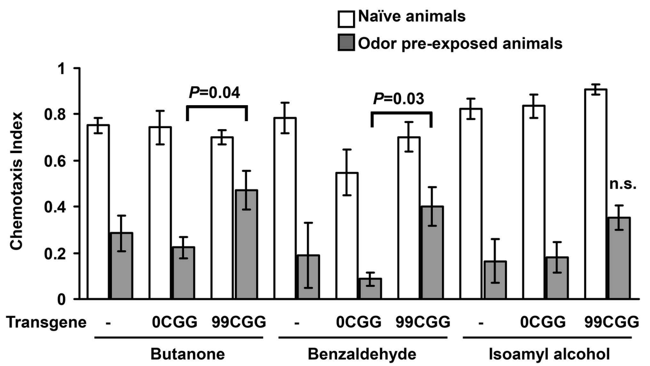
Naïve animals
Odor pre-exposed animals
Chemotaxis Index
1
0.8
0.6
0.4
0.2
0
P=0.04
P=0.03
n.s.
Transgene
-
0CGG
99CGG
-
0CGG
99CGG
-
0CGG
99CGG
Butanone
Benzaldehyde
Isoamyl alcohol

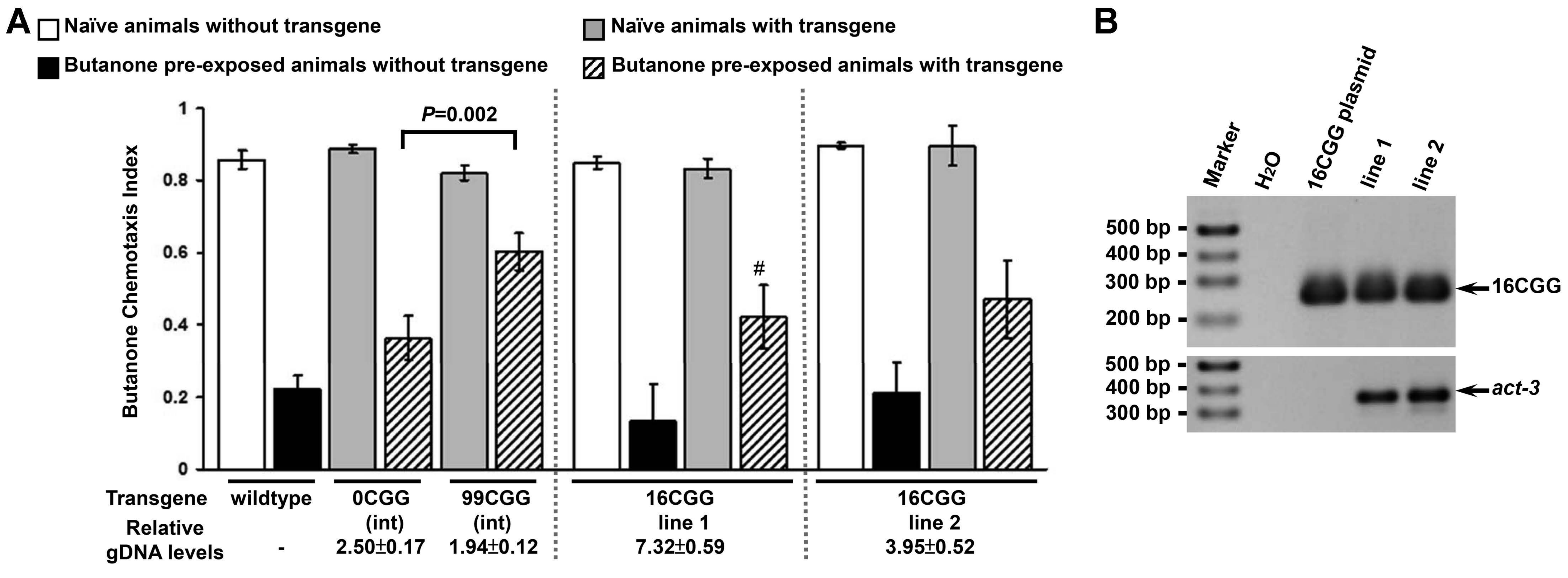

A
Naïve animals without transgene
Naïve animals with transgene
Butanone pre-exposed animals without transgene
Butanone pre-exposed animals with transgene
Butanone Chemotaxis Index
1
0.8
0.6
0.4
0.2
0
P=0.002
#
Transgene
wildtype
0CGG (int)
99CGG (int)
16CGG line 1
16CGG line 2
Relative gDNA levels
-
2.50±0.17
1.94±0.12
7.32±0.59
3.95±0.52
B
Marker
H2O
16CGG plasmid
line 1
line 2
500 bp
400 bp
300 bp
200 bp
16CGG
500 bp
400 bp
300 bp
act-3
